# The Chromosome Number and rDNA Loci Evolution in *Onobrychis* (Fabaceae)

**DOI:** 10.3390/ijms231911033

**Published:** 2022-09-20

**Authors:** Gulru Yucel, Alexander Betekhtin, Evren Cabi, Metin Tuna, Robert Hasterok, Bozena Kolano

**Affiliations:** 1 Plant Cytogenetics and Molecular Biology Group, Faculty of Natural Sciences, Institute of Biology, Biotechnology and Environmental Protection, University of Silesia in Katowice, 40-032 Katowice, Poland; 2 Department of Agricultural Biotechnology, Faculty of Agriculture, Ondokuz Mayis University, Samsun 55200, Turkey; 3 Department of Biology, Institute of Natural and Applied Sciences, Tekirdag Namik Kemal University, Tekirdag 59030, Turkey; 4 Department of Biology, Faculty of Arts and Sciences, Tekirdag Namik Kemal University, Tekirdag 59030, Turkey; 5 Department of Field Crops, Faculty of Agriculture, Tekirdag Namik Kemal University, Tekirdag 59030, Turkey

**Keywords:** *Onobrychis*, chromosome number, dysploidy, polyploidisation, phylogeny, fluorescence in situ hybridisation, rDNA loci

## Abstract

The evolution of chromosome number and ribosomal DNA (rDNA) loci number and localisation were studied in *Onobrychis* Mill. Diploid and tetraploid species, as well as two basic chromosome numbers, *x* = 7 and *x* = 8, were observed among analysed taxa. The chromosomal distribution of rDNA loci was presented here for the first time using fluorescence in situ hybridisation (FISH) with 5S and 35S rDNA probes. *Onobrychis* species showed a high polymorphism in the number and localisation of rDNA loci among diploids, whereas the rDNA loci pattern was very similar in polyploids. Phylogenetic relationships among the species, inferred from nrITS sequences, were used as a framework to reconstruct the patterns of basic chromosome number and rDNA loci evolution. Analysis of the evolution of the basic chromosome numbers allowed the inference of *x* = 8 as the ancestral number and the descending dysploidy and polyploidisation as the major mechanisms of the chromosome number evolution. Analyses of chromosomal patterns of rRNA gene loci in a phylogenetic context resulted in the reconstruction of one locus of 5S rDNA and one locus of 35S rDNA in the interstitial chromosomal position as the ancestral state in this genus.

## 1. Introduction

The genus *Onobrychis* Mill. (Fabaceae) comprises approximately 162 perennial and annual species distributed in temperate zones of North America, the Middle East and Europe [1,2]. The majority of *Onobrychis* species are restricted to Iran, Anatolia and Caucasus, suggesting that these areas were the centre of diversity of the genus [3]. Sirjaev [4], based on floral characteristics, divided the genus into two subgenera: *Onobrychis* Mill (including sections *Onobrychis*, *Dendrobrychis* DC., *Lophobrychis* Hand.-Mazt., *Hemicyclobrychis* (Širj.) Rech.f.) and *Sisyrosema* Bunge (including sections *Anthyllium* Nab., *Afghanicae* Širj., *Heliobrychis* Bunge ex Boiss., *Hymenobrychis* DC.). Recent molecular phylogenetic analyses indicate that *Onobrychis* is a monophyletic taxon and can be further divided into two main evolutionary lineages, each corresponding to the redefined subgenus *Onobrychis* and *Sisyrosema* [5].

Among *Onobrychis*, *O. viciifolia* (sainfoin) is relatively widespread and well known as a cultivated species, mainly used as a forage crop or as a valuable pollen and nectar source for honey production. This crop has a few agronomically useful characteristics, such as drought and cold tolerance and nitrogen fixation [2]. Although the agronomical value of *O. viciifolia* has been emphasised in the literature, the genomic structure of sainfoin and its cultivated and wild relatives remains largely unexplored [2,5], and exact phylogenetic relationships in the genus are unclear. Therefore, the potential utilisation of wild genetic resources in *O. viciifolia* breeding programmes is limited.

Nuclear genome size, a fundamental biological character, was estimated until now only for one *Onobrychis* representative, *O. vicifolia*, which possessed 1C DNA = 1.25 pg (the amount of DNA in the unreplicated haploid nucleus) [1]. To date, cytogenetic analyses in *Onobrychis* have been mainly restricted to chromosome number reports. Few studies on karyotype structure and evolution have shown that *Onobrychis* species have, in general, short and mostly metacentric or submetacentric chromosomes [6]. Two basic chromosome numbers, *x* = 7 and *x* = 8, and species on two ploidy levels, diploids (2*n* = 2*x* = 14 or 16) and tetraploids (2*n* = 4*x* = 28 or 32) were reported in the genus [6,7,8,9,10].

Two mechanisms, dysploidy and polyploidy, are responsible for the polymorphisms in chromosome number in most analysed plant genera [11,12,13]. No common trends in the evolution of chromosome numbers among analysed taxa were observed; however, polyploidy and descending dysploidy seem to be most often reported [14,15,16,17]. A serious weakness of the analyses of *Onobrychis* karyotypes is the paucity of chromosome markers, which has limited the identification of individual chromosomes and makes a comparative analysis very difficult. Fluorescence in situ hybridisation (FISH) often provides informative chromosome markers with a single copy and repetitive sequences as DNA probes [18,19,20]. Ribosomal DNA (rDNA)-based sequences are the markers of the first choice in comparative analyses of chromosomes, especially in wild and neglected plant species [21,22,23]. The genes encoding 35S ribosomal RNA (18S-5.8S-25S rRNA) and 5S rRNA usually are present in separate, unlinked loci as tandem arrays in the genomes of angiosperms [24]. In most plant genomes, low numbers of rDNA loci were observed. The median plant karyotype has two loci of 5S rDNA in the interstitial chromosome regions and two loci of 35S rDNA in the subterminal position [25]. In many cases, rDNA sequences were shown to be useful chromosome markers that enable the unambiguous identification of some chromosomes in karyotypes of species from various angiosperm families [26,27,28]. In addition, among diploid *Hedysarum* species (2*n* = 14), the genus closely related to *Onobrychis*, the application of 5S and 25S rDNA probes enables the identification of two to four out of seven chromosomes [29]. The genes encoding 35S and 5S rRNA consist of evolutionary highly conserved coding sequences and variable noncoding regions [30]. rDNA sequences are used not only as universal chromosomal markers but also as phylogenetic markers [31,32]. In particular, the nuclear ribosomal internal transcribed spacer (nrITS) within the 35S rDNA unit has often been used for phylogenetic analyses at the species, genus, and family levels [32,33,34]. Combining cytogenetic and phylogenetic approaches allows a more precise interpretation of cytogenetic data in a phylogenetic context.

This research aimed to study the patterns of chromosome number and rDNA loci evolution in *Onobrychis* species and produce more informative karyotypes for comparative analyses. The chromosome number was determined for 29 species, two of which were reported here for the first time. The number and chromosomal localisation of rDNA loci (35S and 5S rDNA) were first time analysed in 25 *Onobrychis* species. The phylogenetic relationships among diploid and polyploid species inferred from analyses of nrITS sequences were used to reconstruct the ancestral states of the chromosome number and 35S and 5S rDNA loci number in *Onobrychis*.

## 2. Results

### 2.1. Chromosome Number

**Figure 1:**
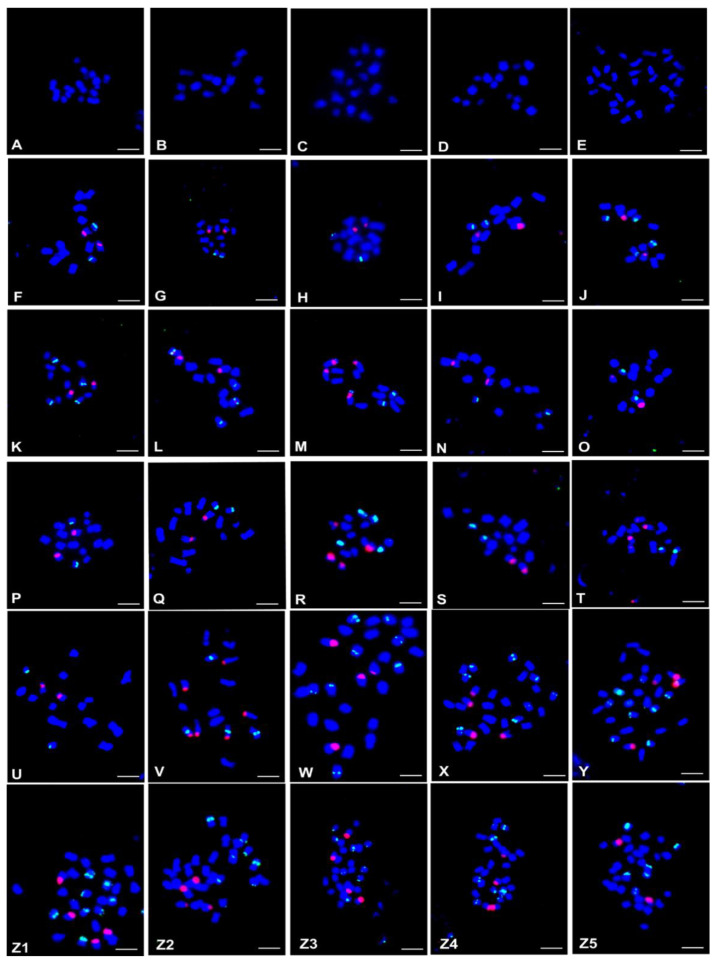
Chromosome number and distribution of rDNA loci in diploid and polyploid *Onobrychis* species. Fluorescence in situ hybridisation was performed with 5S rDNA probe (green fluorescence) and 35S rDNA probe (red fluorescence). (**A**) *O. sternohiza*, (**B**) *O. humilis*, (**C**) *O. michauxii*, (**D**) *O. gaubae*, (**E**) *O. altissima*, (**F**) *O. grandis*, (**G**) *O. caput-galli*, (**H**) *O. ptolemaica*, (**I**) *O. hypargyrea*, (**J**) *O. gracilis*, (**K**) *O. supina*, (**L**) *O. megataphros*, (**M**) *O. alba subsp. laconica*, (**N**) *O. sintenisii*, (**O**) *O. vassilczenkoi*, (**P**) *O. chorossanica*, (**Q**) *O. kachetica*, (**R**) *O. persica*, (**S**) *O. radiata*, (**T**) *O. vaginalis*, (**U**) *O. iberica*, (**V**) *O. crista-galli*, (**W**) *O. viciifolia*1 (PI170583), (**X**) *O. viciifolia* (PI200872), (**Y**) *O. arenaria*, (**Z1**) *O. transcaucasica*, (**Z2**) *O. cyri*, (**Z3**) *O. biebersteinii*, (**Z4**) *O*. *inermis*, (**Z5**) *O. subacaulis.* All bars: 5 µm.

Chromosome number was analysed for 30 accessions representing 29 *Onobrychis* species. The analysed species revealed two different basic chromosome numbers, *x* = 7 (17 species) and *x* = 8 (12 species). Ten *x* = 7 species were diploids with the chromosome number 2*n* = 2*x* = 14 and seven were tetraploids with 2*n* = 4*x* = 28. Eleven *x* = 8 diploids showed chromosome number 2*n* = 2*x* = 16, and one was a tetraploid with 2*n* = 4*x* = 32 (Table 1; Figure 1A–Z5). The chromosome number was newly determined for two species: *O. stenorhiza* (2*n* = 2*x* = 14) and *O. vaginalis* (2*n* = 2*x* = 16, Table 1; Figure 1A,T).

### 2.2. Number and Localisation of rDNA Loci

The number and localisation of the rDNA loci were determined using FISH with 25S and 5S rDNA probes. The rDNA number and localisation are reported for 17 diploid and seven tetraploid *Onobrychis* species. Except for *O. viciifolia*, only one accession per species was analysed in this study. The number and chromosomal localisation of 5S rDNA and 35S rDNA loci are demonstrated in Figure 1 and schematically summarised in Table 1.

Among the diploids, high interspecific polymorphisms of rDNA loci number and localisation were observed, and nine patterns of rDNA loci chromosomal organisation were distinguished (Figure 1F–Z5; Table 1). The number of 35S rDNA loci ranged from one to three (Figure 1), whereas the number of 5S rDNA loci was from one to two per haploid chromosome set in diploids (Figure 1); however, most often (10 species) one locus of 35S rDNA and one locus of 5S rDNA was observed (Table 1; Figure 1F–I,N–Q,S,U). The locus of 35S rDNA in the interstitial chromosomal position was most often observed (14 diploid species; Figure 1F,H–Q,S–U). The subterminal position of 35S rDNA loci was observed in three diploid species (Figure 1G,R,V). In karyotypes of eight diploids, the locus of 5S rDNA was observed exclusively in the subterminal position (Figure 1H,N–Q,S–U). The interstitial localisation of the 5S rDNA locus/loci was found in seven species (Figure 1F,G,I–L,V), whereas two species had both interstitial and subterminal loci of 5S rDNA (Figure 1M,R). Most analysed diploids had 5S and 35S rDNA loci in different chromosome pairs (Figure 1F–L,N–Q,S–U). In karyotypes of three species (*O. megataphros*, *O. alba* subsp. *laconica* and *O. crista-galli*), one or two chromosomes bearing both 35S and 5S rDNA loci were observed (Figure 1L,M,V).

### 2.3. Molecular Phylogenetic Analysis of nrITS

**Figure 2:**
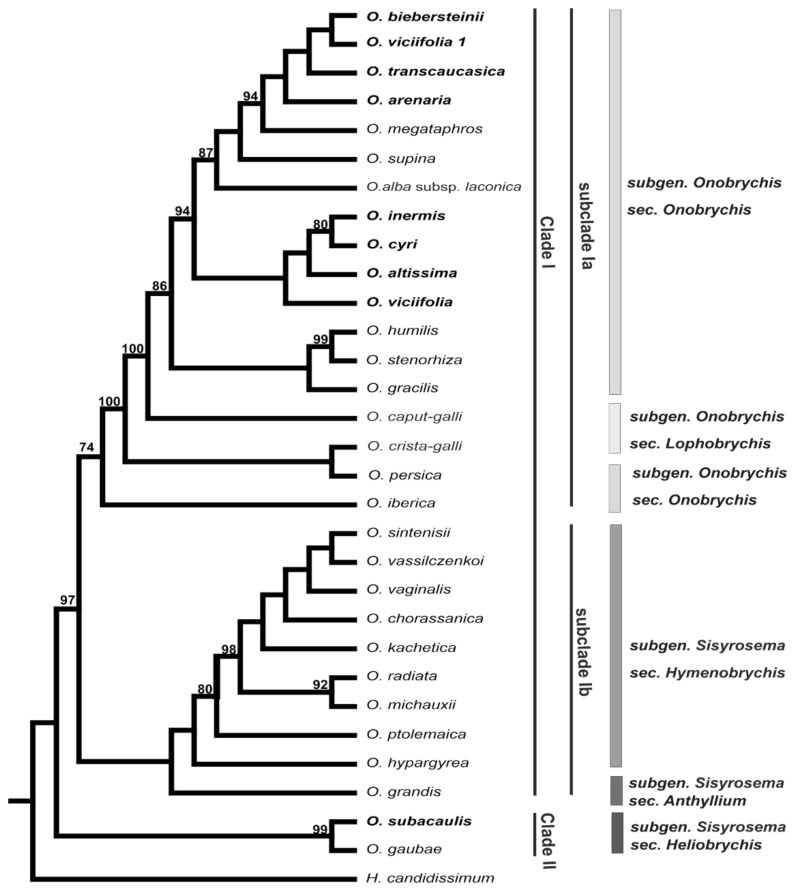
Phylogenetic relationships among analysed *Onobrychis* species based on the nrITS data set. Bootstrap support values are shown above the branches. The tetraploid species are shown in bold. The tree was rooted with *Hedysarum candidissimum*.

Molecular phylogenetic analysis of 30 accessions representing 21 diploid and eight polyploid *Onobrychis* species was performed based on nrITS sequences. The total length of the analysed nrITS DNA regions differed among the analysed species and ranged from 491 to 593 bp. The final alignment was 626 bp long (including gaps), with 91 characters that were potentially parsimony informative. Maximum likelihood (ML) analyses of nrITS datasets assigned all analysed *Onobrychis* species into two main clades (Figure 2). Clade II (BS99) consisted of diploid (*O. gaubae*, 2*n* = 2*x* = 16) and polyploid species (*O. subacaulis*, 2*n* = 4*x* = 32) from subgenus *Sisyrosema* section *Heliobrychis* (Figure 2). Clade I comprised twenty diploids and seven polyploids from two subgenera *Sisyrosema* and *Onobrychis*. Clade I was further divided into two subclades: (i) subclade Ia included species from subgenera *Onobrychis* sections *Onobrychis* and *Lophobrychis*. Among them were eight diploids (*O. megataphros*, *O. supina*, *O. alba subsp. laconica*, *O. humilis*, *O. stenorhiza*, *O. gracilis*, *O. persica* and *O. iberica*) and seven polyploids (*O. biebersteinii*, *O. viciifolia*, *O. transcaucasica*, *O. arenaria*, *O. inermis*, *O. cyri*, *O. altissima*) from section *Onobrychis* and two diploids (*O. caput-galli* and *O. crista-galli*) from section *Lophobrychis*; (ii) subclade Ib included nine diploids from subgenus *Sisyrosema* section *Hymenobrychis* (*O. sintenisii*, *O. vassilczenkoi*, *O. vaginalis*, *O. chorossanica*, *O. kachetica*, *O. radiata*, *O. michauxii*, *O. ptolemaica*, *O. hypargyrea*) and one diploid from the section *Anthyllium* (*O. grandis*; Figure 2). The polyploids in subclade Ia were included in two groups of closely related species. Interestingly, two analysed accessions of *O. viciifolia* belonged to two different groups.

### 2.4. Inferences of the Patterns of Chromosome Number Evolution

**Figure 3:**
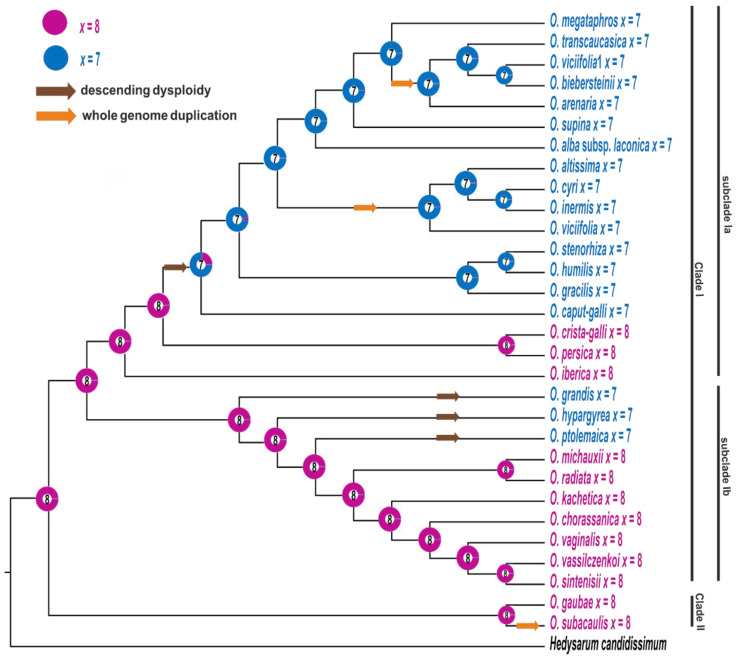
Ancestral character state reconstruction of the basic chromosome numbers of the analysed species of *Onobrychis*. The chromosome numbers have been mapped on the ML tree of nrITS sequences using the maximum likelihood method implemented in ChromEvol 2.0 software. The tree was rooted with *Hedysarum candidissimum*.

The basic chromosome number *x* = 8 was recovered as the ancestral state for all studied *Onobrychis* species and as the ancestral state for all distinguished clades and subclades (Figure 3). Although in subclade Ib most of the species had *x* = 8, three events of descending dysploidy (from *x* = 8 to *x* = 7) were inferred (in *O. ptolemaica*, *O. hypargyrea* and *O. grandis* evolutionary lineages). In subclade Ia, only three diploid species had *x* = 8 (*O. crista-galli*, *O. persica*, *O. iberica*), while the remaining species had *x* = 7, and only one event of descending dysploidy was retrieved. Three events of chromosome number duplication were reconstructed: (i) in the evolutionary lineage of *O. subacaulis* (clade II); (ii) for common ancestor of *O. arenaria*, *O. transcaucasia* and *O. bibersteinii* and one accession of *O. viciifolia* and (iii) for common ancestor of *O. altissima*, *O. cyri*, *O. inermis* and the second accession of *O. viciifolia* (Figure 3 and Figure S1).

### 2.5. Evolutionary Patterns of the rDNA Loci Chromosomal Organisation

**Figure 4:**
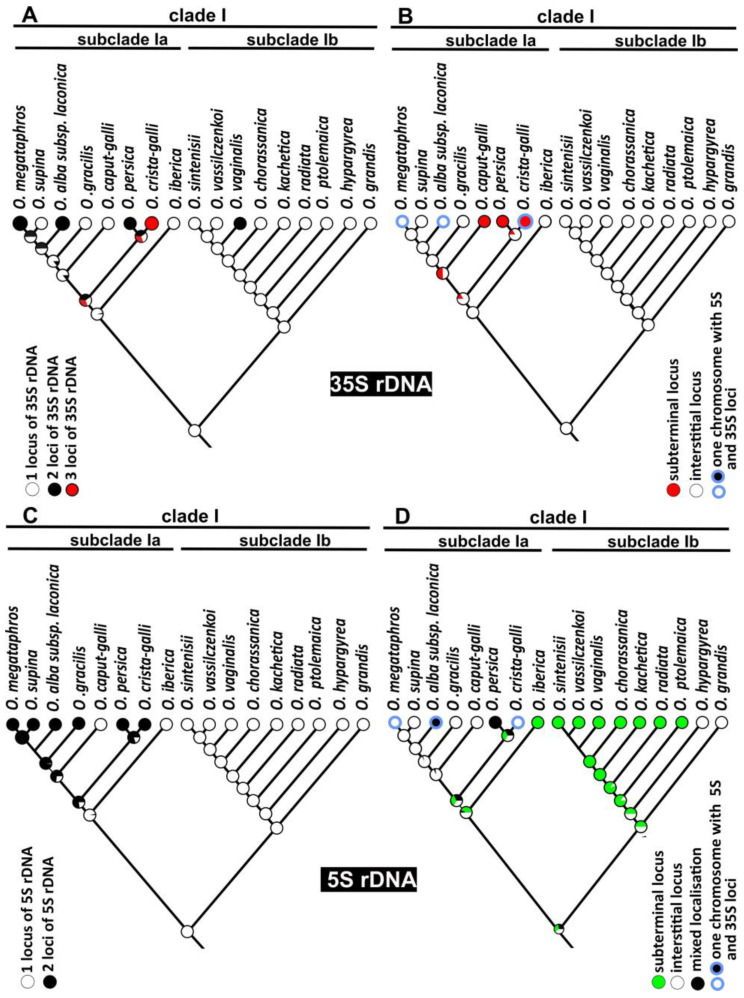
Ancestral character state reconstruction of the rDNA locus number and localisation for *Onobrychis* species. The numbers and localisation of the rDNA loci were mapped onto the ML tree of the nrITS sequences using maximum likelihood methods. (**A**) The number of 35S rDNA loci. (**B**) Localisation of 35S rDNA loci. (**C**) The number of 5S rDNA loci. (**D**) Localisation of 5S rDNA loci. The tree was rooted with *Hedysarum candidissimum*, which was subsequently removed from the figure.

The number and localisation of rDNA loci were analysed using FISH for diploids from clade I. The obtained data were mapped on the ML phylogenetic tree using the maximum likelihood reconstruction methods. The analyses resulted in the reconstruction of one locus of 35S rDNA in the interstitial position in the chromosome and one locus of 5S rDNA as an ancestral state of clade I and both distinguished subclades Ia and Ib (Figure 4). Unlike for the 35S rDNA locus, the ancestral state of chromosomal localisation of the 5S rDNA loci was ambiguously retrieved. The ancestral state of 35S rDNA locus number and localisation was observed in most species in the Ib clade. Gain of the 35S rDNA locus accompanied speciation of only one species, *O. vaginalis* (Figure 4A). In subclade Ib, all species also showed an ancestral state, one locus of 5S rDNA. In karyotypes of most species from this subclade, this locus was in the subterminal position (Figure 4C). The analyses suggested one or two repositioning events of 5S rDNA loci during the evolution of the subclade Ib (*O. hypargyrea* and *O. grandis*; Figure 4B). One interstitial locus of 35S rDNA and one locus of 5S rDNA were also reconstructed as an ancestral state for subclade Ia. However, the patterns of rDNA loci evolution were more complicated in this subclade. The ancestral state, one locus of 35S rDNA was observed in four out of eight species (*O. iberica*, *O. supina*, *O. caput-gali* and *O. gracilis*; Figure 4C). The evolution of the remaining species was accompanied by a gain of one locus (*O. persica*, *O. alba* subsp. *laconica* and *O. megataphorus*) or two loci (*O. crista-galli*) of 35S rDNA (Figure 4A). The diversification of these species was also accompanied by at least two events of 35S rDNA loci repositioning from the interstitial (ancestral state) to the subterminal localisation (Figure 4B). The ancestral number, one locus of 5S rDNA, was present only in the karyotype of two species, *O. iberica* and *O. caput-galli* from subclade Ia. The gain of 5S rDNA loci was retrieved for the common ancestor of most diploids from subclade Ia (except *O. iberica*, which has the ancestral number of loci), followed by the loss of 5S rDNA loci during speciation of *O. caput-galli* (Figure 4C). Additionally, repositioning of 5S rDNA locus may also be suggested to accompany the speciation of *O. iberica* (species with subterminal 5S rDNA locus) or the evolution of all other species from subclade Ia (at least one locus of 5S rDNA in interstitial localisation). Moreover, three diploids (*O. megataphros*, *O. crista-galli* and *O. alba* subsp. *laconica*) that had duplicated (or triplicated) numbers of both rDNA loci included in their karyotypes a chromosome bearing both 35S and 5S rDNA loci (Figure 4).

## 3. Discussion

The analyses of chromosome number and rDNA loci chromosomal organisation in the phylogenetic framework enable insight into the trends in chromosomal evolution that accompany or follow diversification and speciation in plants, especially in wild or non-model taxa such as *Chenopodium*, *Crepis*, *Prospero* and *Allium* [18,35,36]. Although recently, *O. vicifolia* has started to attract more attention as a fodder crop, relatively little research has been conducted on *Onobrychis* genome structure and evolution. In earlier reports, two basic chromosome numbers, *x* = 7 and *x* = 8, were observed among analysed representatives of this genus [6,7]. The same basic chromosome numbers were also reported for the closely related genus *Hedysarum* [37]. More than one basic chromosome number was reported in several other genera from the Fabaceae family (e.g., *Vicia*, *Phaseolus* and *Lotus*) [38,39,40] as well as in other plant families, e.g., in *Crepis* from Asteraceae or *Passiflora* from Passifloraceae [16,41]. Relatively many reports were published regarding chromosome numbers in *Onobrychis.* However, the chromosome counts for several species differed among publications, e.g., for the same species, both diploid and tetraploid chromosome numbers were reported (e.g., *O. crista-galli* and *O. caput-galli*) [7,42,43] (Table S1 [6,7,8,9,10,37,43,44,45,46,47,48,49,50,51,52,53,54,55,56]). Different basic chromosome numbers were reported for some *Onobrychis* species, e.g., *x* = 7 and *x* = 8 for *O. ptolemaica* [7,10] (Table S1). Intraspecific polymorphisms in chromosome number concerning ploidy levels were published in several different genera, e.g., *Prospero autumnale*, *Deschampsia cespitosa* *sensu lato* and *Crepis vesicaria* [57,58,59]. Few reports also showed that some species consist of cytotypes which differ in basic chromosome number, which could be caused by aneuploidy, e.g., trisomy of one chromosome pair in *Amaranthus caudatus* [59] and dysploidy in, e.g., *Prospero atumnale* complex [36]. However, technical issues could also cause incongruence between different reports. *Onobrychis* species have relatively small chromosomes, and most of the chromosome counts were based on simple techniques like Feulgen or acetoorcein/acetocarmine staining (e.g., [10,37]). In karyotypes with small and numerous chromosomes, these methods could not allow precise identification of specific chromosomes and assigning them into homologous pairs. In the case of *Onobrychis*, the differences between chromosome reports could also be attributed to the highly complex taxonomy of this genus. Different phylogenetic approaches to species delimitation resulted in varying numbers of recognised species [1]. Thus, all the analyses in our study were carried out on the same individuals of the species, enabling optimal correlation of the molecular and cytogenetic data.

Phylogeny based on the biparentally inherited nrITS of analysed *Onobrychis* species was largely congruent with previously published results [5,60]. In the present study, all analysed species from subgenus *Onobrychis* were included in one subclade, as earlier shown, based on nrITS and plastid markers [5,60,61]. The second subgenus *Sisyrosema*, previously reported as monophyletic [5,60], was recovered in two separate clades in the present study. This incongruence might be due to technical reasons because each analysis was based on different sets of species. In every report published up to date, the analysed species accounted for 20% to 40% of all species included in this genus [5,60,61]. Moreover, some nodes in nrITS phylogram in subgenus *Sisyrosema* were weakly supported. The two accessions of tetraploid *O. vicifolia* from subgenus *Onobrychis* were included in two different evolutionary lineages. One of these accessions was a cultivated form, whereas the other was a wild plant. Wild and domesticated plants are shaped by evolutionary responses to different selection pressures, which may lead to genetic diversification between these species forms [62,63]. The origin of tetraploid *O. viciifolia* (allo- or autopolyploidy) and its genome composition are unknown. In allopolyploids, 35S rDNA often undergoes homogenisation towards either the maternal or paternal ribotype, and both scenarios are well-documented [34,58,64]. In addition, the extent of homogenisation and conversion can differ between older and recently formed polyploid accessions of the same species [65,66], and thus, the presence of *O. vicifolia* lineages that differed in ribotype variants might suggest multiple origins of this tetraploid similar to the case in other polyploids [67,68].

The analyses of basic chromosome numbers in the phylogenetic context showed that *x* = 8 was reconstructed as ancestral for all analysed species as well as for species from subgenera *Sisyrosema* and *Onobrychis;* however, the patterns of basic chromosome number evolution were different in these two subgenera. In both of them, species with *x* = 8 and *x* = 7 were revealed, but most *Sisyrosema* subgenus species have *x* = 8, whereas those belonging to the subgenus *Onobrychis* have *x* = 7. One event of descending dysploidy at a relatively deep node was inferred in the subgenus *Onobrychis.* In contrast, three independent events of descending dysploidy were reconstructed at the tips of the tree in the subgenus *Sisyrosema.* The recurrent events of dysploidy were recovered for several genera. Often the derived basic chromosome number appeared several times during the diversification and evolution of a genus, e.g., in *Crepis* and *Artemisia* [16,69]. On the other hand, in taxa, such as *Helianthemum* or *Chenopodium*, the basic chromosome number is a genus-specific feature [18,70].

Three events of whole genome duplication were reconstructed for the analysed *Onobrychis*, one for the species with *x* = 8 (subgenus *Sisyrosema*) and two for the species with *x* = 7 (subgenus *Onobrychis*). In subgenus *Onobrychis*, the reconstructed polyploidisation events might suggest a common tetraploid ancestor for *O. transcaucasia*, *O. bibersteinii* and cultivated *O. viciifolia*, as well as a common tetraploid ancestor for *O. altissima*, *O. inermis*, *O. cyri* and the wild accession of *O. viciifolia*. Since only one type of nrITS was amplified from the polyploid *Onobrychis*, this only suggests that the tetraploids, which group together, probably share one parental taxon. Thus, the data do not allow inferences of independent or common origins of analysed groups of polyploids. Moreover, Hayot Carbonero et al. [1] suggest that the *Onobrychis* taxonomy is over-complicated by the existence of synonyms and spurious subspecies. For example, *O. pyrenaica*, *O. altissima*, *O. arenaria*, *O. inermis* and *O. montana* might all be synonyms for *O. viciifolia*.

In some cases, rDNA loci are robust chromosome markers, allowing comparative analyses of karyotypes, especially of wild and neglected species [22,71,72,73]. In angiosperms, most often 35S rDNA loci are placed in the subterminal while most 5S rDNA loci are in the interstitial region of the chromosome [21,74]. In contrast, the chromosomal distribution of these loci is usually opposite, with 35S rDNA localised interstitial and 5S rDNA terminal in *Onobrychis.* Regarding diploid *Onobrychis*, both these from the subclade Ia and Ib showed diverse patterns of rDNA loci number and distribution. While in subclade Ib relatively few events of rDNA loci reorganisation (one duplication of 35S rDNA and one or two repositionings of 5S rDNA loci) were retrieved, in subclade Ia nearly every species showed different patterns of rDNA loci organisation with duplication or even triplication of their number being the most common. The patterns of rDNA loci evolution often differ between evolutionary lineages [75]. The genera such as *Trifolium*, *Iris* and *Citrullus* show high interspecific polymorphisms in rDNA loci chromosomal patterns [76,77,78]. On the other hand, such taxa as *Chenopodium* and *Daucus* may exhibit little or no variation in this respect [18,79]. Usually, 35S rDNA loci tend to be more variable [21], but in analysed *Onobrychis*, chromosomal distribution of both rDNA loci seems equally polymorphic. The repositioning of rDNA loci might indicate chromosome rearrangements such as translocation or inversion, or result from transposon-mediated transposition events [80,81,82,83]. The rDNA loci consist of arrays of evolutionarily conserved repeats and mechanisms based on recombination (e.g., unequal recombination, illegitimate recombination) may play a role in the evolution of high variability in rDNA patterns even among closely related species [84,85].

A chromosome bearing both types of rRNA gene loci was present in karyotypes of three species from subclade Ia. The evolution of these species was accompanied by duplication of 5S or/and 35S rDNA loci. Thus the most plausible explanation of the new pattern seems to be the insertion of a complementary rDNA array into chromosomes which already possess 35S or 5S rDNA locus. In karyotypes of species with multiple rDNA loci, the localisation of 5S and 35S rDNA sites in one chromosome was relatively often observed (nearly 53% of analysed species), while in species with a single locus of 5S and 35S rDNA, this pattern was rare (approximately 13% [74]). The insight into the rDNA organisation in the diploid ancestors is a prerequisite to hypothesising the evolution of rDNA in their derived polyploids. Although the origin of tetraploid *Onobrychis* is unknown, eliminating at least one 35S rDNA loci might be suggested in *O. subacaulis*, a tetraploid with only one locus of these genes. All analysed tetraploids from subgenus *Onobrychis* showed the same pattern of rDNA loci distribution, thus supporting the hypothesis of their close relationship (one or both common or close ancestors). However, rDNA loci in polyploids often undergo various reorganisations. Thus in many cases, the polyploidy does not reveal the expected additive patterns of the putative parental taxa. This phenomenon could be observed as the loss, gain or repositioning of rDNA loci [11,46,86,87,88].

## 4. Materials and Methods

### 4.1. Plant Material

Thirty accessions representing 29 *Onobrychis* species were analysed. Three studied accessions represented the only cultivated species (*O. viciifolia*, *O. arenaria* and *O. transcaucasica*) of the genus. Information about the material is listed in Table 2. Analysed material was grown from seeds in a greenhouse facility of the University of Silesia in Katowice, Poland. The analysed species belong to two subgenera: *Onobrychis* and *Sisyrosema*. The traditional taxonomy, according to Sirjaev [4], Grossheim [89] and Rechinger [90], were used in this study (Table S2). Vouchers are deposited at the Herbarium KTU (University of Silesia, Katowice, Poland).

### 4.2. DNA Amplification and Sequencing

Total genomic DNA was isolated from fresh, healthy leaf tissue using the modified cetyltrimethylammonium bromide (CTAB) method [91]. DNA concentration was measured using an ND-1000 spectrophotometer (peqLab, Erlangen, Germany). The nrITS region was amplified using a primer pair anchored in 18S rDNA and 25S rDNA (18S dir (5′-CGTAACAAGGTTTCCGTAGG-3′) and 25S com (5′-AGCGGGTAGTCCCGCCTGA-3′) [92]). PCR reaction mixture contained 0.4 μM of each primer (Genomed, Warsaw, Poland), 0.2 mM of each deoxynucleoside triphosphate (dNTP; Sigma-Aldrich, Steinheim, Germany), 50 ng DNA, 1 × PCR buffer (including 1.5 mM MgCl_2_) and 1 U Taq DNA polymerase (Sigma-Aldrich, Steinheim, Germany). Polymerase chain reaction was carried out using the GeneAmpPCR system 9700 thermocycler (Applied Biosystems, Waltham, MA, USA). The PCR reaction was performed with an initial denaturation at 94 °C for 3 min, followed by 40 cycles of 30 s at 94 °C, 1 min at 50 °C and 45 s at 72 °C, with a final elongation step of 5 min at 72 °C. PCR products were treated with *Escherichia coli* exonuclease I and FastAP thermosensitive alkaline phosphatase (Thermo Fisher Scientific, Waltham, MA, USA) according to the manufacturer’s protocol. Sequencing was performed using BigDye Terminator v3.1 technology (Applied Biosystems) and 3730xl DNA Analyzer (Applied Biosystems) in a commercial facility (Macrogen, Amsterdam, Netherlands). All sequences were deposited in GenBank, and accession numbers are presented in Table 2.

### 4.3. Sequence Alignment and Phylogenetic Analyses

Sequences were assembled using DNA Baser 3 (Heracle BioSoft S.R.L., Pitesti, Romania). Multiple sequence alignments for all datasets were performed 20 times using webPRANK [93], and MergeAlign [94] was subsequently used to obtain a consensus on the multiple sequence alignments. Phylogenetic relationships for nrITS regions using maximum likelihood (ML) analyses as implemented in IQ-TREE were inferred [95]. The significance of the inferred relationships was assessed via bootstrapping with 1000 replicates. The most appropriate model of sequence evolution for the ML analyses was determined using the Bayesian information criterion as implemented in IQ-TREE. The best-fit model was TNe + G4 for the nrITS data set. *Hedysarum candidissimum* Freyn. was used as an outgroup (GenBank accession number GQ246080). The resulting phylogenetic tree was created using FigTree v.1.3.1 [96]. Bootstrap support values below 75 from the figures were excluded.

### 4.4. Chromosome Preparation and Fluorescence In Situ Hybridisation

The chromosome preparations were made as described previously [97]. The 5S rDNA monomer that had been isolated from (clone pTa794 [98]) and labelled with digoxigenin-11-dUTP (Roche, Basel, Switzerland) to detect 5S rDNA loci was used. A 2.3-kb fragment of the 25S rDNA coding region of *Arabidopsis thaliana* [99] labelled with tetramethyl-rhodamine-5-dUTP (Roche) was used to detect the 35S rDNA loci. The probe labelling and FISH followed the Jenkins and Hasterok protocol [100]. The hybridisation mixture consisted of 50% deionised formamide, 10% dextran sulphate, 2x SSC, 0.5% SDS (sodium dodecyl sulphate) and labelled probes (100 ng of each probe per slide). Hybridisation was conducted for 48 h at 37 °C in a humid chamber. Post-hybridisation washes (10% deionised formamide in 0.1x SSC at 42 °C; stringency 76%) were followed by the immunodetection of digoxigenated probes using FITC-conjugated anti-digoxigenin antibodies (Roche). The slides were mounted in Vectashield (Vector Laboratories, Newark, CA, USA) containing 2.5 ng/µL of DAPI (4′,6-diamidino-2-phenylindole dihydrochloride). All images were acquired using a Zeiss AxioImager.Z.2 fluorescent microscope equipped with an AxioCam HMr camera (Zeiss, Oberkochen, Germany). The images were processed uniformly using ZEN 2.3 Pro (Zeiss). FISH experiments with 35S and 5S rDNA probes were conducted for 24 species, and the slides after FISH were used for chromosome counting. Chromosome counts were performed for five more species (*O. humilis*, *O. stenorhiza*, *O. altissima*, *O. gaubae*, and *O. michauxii*), based on slides stained with DAPI. FISH was not applied for these species due to an insufficient amount of material for analyses.

### 4.5. Inferences of the Patterns of Evolution of Chromosome Number and rDNA Loci Number and Localisation

The phylogram resulting from the ML analysis (branch length information included) was used to infer the evolution of the basic chromosome number and rDNA loci number and localisation. The analyses of chromosome numbers using maximum likelihood as implemented in ChromEvol 2.0. software were performed [101]. The best-fit model was tested using an AIC test. The maximum likelihood analyses were performed under the CONST_RATE model as implemented in ChromEvol 2.0. For the best-fitted model, the analyses were rerun with fixed parameters to those optimised in the first run and using 10,000 simulations to compute the expected number of changes along each branch and the ancestral basic chromosome numbers at nodes. The analyses of rDNA loci evolution using maximum likelihood (for discrete characters) as implemented in Mesquite 2.74 were performed. Four characters were analysed separately: (i) the number of 35S rDNA loci, (ii) the localisation of 35S rDNA loci, (iii) the number of 5S rDNA loci and (iv) the localisation of 5S rDNA loci [102].

## 5. Conclusions

Both analysed *Onobrychis* subgenera show different patterns regarding the evolution of both chromosome number and rDNA loci chromosomal organisation. Descending dysploidy and polyploidisation seem to be mechanisms which shape their chromosome number. Several events of rDNA locus repatterning involving the gains and repositioning of 35S and 5S rDNA loci were proposed to explain their distribution in extant *Onobrychis* diploids. Our research should serve as the foundation for more detailed analyses of the *Onobrychis* genomes using more chromosomal markers representing various repetitive DNA families. Identifying putative parental species is necessary to understand better evolutionary genome changes that accompanied speciation of the domesticated and wild tetraploids in the genus.

## Figures and Tables

**Table 1:** The number of chromosomes and the number and localisation of rDNA loci in analysed *Onobrychis* species.

Taxon	2*n*	rDNA Loci Number and Localisation *		
		35S rDNA	5S rDNA	
Subgenus *Onobrychis*				
*O. alba* subsp. *laconica*	14	2I	1T, 1I	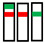
*O. caput-galli*	14	1T	1I	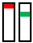
*O. crista-galli*	16	3T	2I	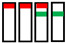
*O. gracilis*	14	1I	2I	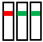
*O. humilis*	14	-	-	-
*O. iberica*	16	1I	1T	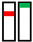
*O. megataphros*	14	2I	2I	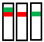
*O. persica*	16	2T	1I, 1T	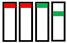
*O. stenorhiza*	14	-	-	-
*O. supina*	14	1I	2I	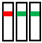
*O. altissima*	28	-	-	-
*O. biebersteinii*	28	2T	2T, 2I	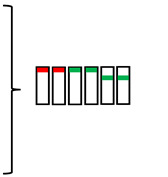
*O. viciifolia* 1	28	2T	2T, 2I	
*O. viciifolia* 2	28	2T	2T, 2I	
*O. transcausica*	28	2T	2T, 2I	
*O. arenaria*	28	2T	2T, 2I	
*O. inermis*	28	2T	2T, 2I	
*O. cyri*	28	2T	2T, 2I	
**Subgenus** ***Sisyrosema***				
*O. chorossanica*	16	1I	1T	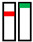
*O. grandis*	14	1I	1I	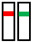
*O. gaubae*	16	-	-	-
*O. hypargyrea*	14	1I	1I	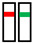
*O. kachetica*	16	1I	1T	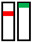
*O. michauxii*	16	-	-	-
*O. sintenisii*	16	1I	1T	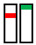
*O. vassilczenkoi*	16	1I	1T	
*O. vaginalis*	16	2I	1T	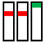
*O. radiata*	16	1I	1T	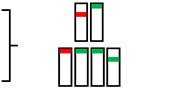
*O. ptolemaica*	14	1I	1T	
*O. subacaulis*	32	1T	2T, 1I	

* T—subterminal locus, I—interstitial locus.

**Table 2:** General characteristics of the analysed taxa and the GenBank accession numbers of the sequences obtained in this study.

Species	USDA * Collection Number	Voucher	GeneBank Accession
*O. biebersteinii* Sirj.	PI 227377	KTU154634	OP288059
*O. viciifolia* Scop. (1)	PI 1705831 #	KTU154645	OP288048
*O. viciifolia* Scop. (2)	PI 200872	KTU154636	OP288055
*O. transcaucasica* Grossh.	PI 273771 #	KTU154640	OP288065
*O. arenaria* (Kit.) DC.	PI 273743 #	KTU154639	OP288054
*O. megataphros*	PI 301107	-	OP288064
*O. supina* (Vill.) DC.	PI 383721	KTU154646	OP288047
*O. alba* (Waldst. & Kit.) Desv. subsp. *laconica* (Boiss.) Hayek	W6 19337	KTU154647	OP288049
*O. inermis* Steven	W617870	KTU154654	OP288053
*O. cyri* Grossh.	W6 17800	KTU154635	OP288070
*O. altissima Grossh.*	PI 325448	-	OP288067
*O. humilis (Loefl.) G. Lopez*	PI 319054	-	OP288046
*O. stenorhiza* D.C	PI 319056	-	OP288066
*O. gracilis* Besser	W6 19496	KTU154642	OP288050
*O. caput-galli* (L.) Lam.	PI 205304	KTU154659	OP288056
*O. persica* Sirj. & Rech.f.	PI 380946	KTU154638	OP288071
*O. crista-galli* (L.) Lam.	PI 227040	KTU154651	OP288068
*O. iberica* Grossh.	PI 219602	-	OP288058
*O. sintenisii* Bornm.	PI 314100	KTU154632	OP288057
*O. vassilczenkoi* Grossh.	PI 678913	KTU154641	OP288063
*O. vaginalis* C.A. Mey.	PI 325444	KTU154633	OP288051
*O. chorossanica* Bunge ex Boiss.	PI 314160	KTU154658	OP288061
*O. kachetica* Boiss. & Buhse	PI 314469	KTU154649	OP288062
*O. radiata* (Desf.) M. Bieb.	W6 24111	KTU154650	OP288074
*O. michauxii* D.C.	PI 380945	-	OP288060
*O. ptolemaica* (Delile) DC.	PI 215344	KTU154655	OP288073
*O. hyparygera* Boiss.	PI 383719	KTU154644	OP288052
*O. grandis* Lipsky	PI 440568	KTU154653	OP288072
*O. subacaulis* Boiss.	PI 219930	KTU154643	OP288075
*O. gaubae* Bornm.	PI 380931	-	OP288069

USDA North Central Regional Plant Introduction Station of the US National Plant Germplasm System. # Cultivated form.

## Data Availability

The nucleotide sequences are available in GenBank (http://www.ncbi.nlm.nih.gov/genbank (accessed on 22 August 2022)) under numbers OP288046–OP288075. Other data generated or analysed during this study are available from the corresponding authors upon reasonable request.

## Supplementary Materials

The supporting information can be downloaded at: https://www.mdpi.com/article/10.3390/ijms231911033/s1.
