# Supplementary material for: The Chromosome Number and rDNA Loci Evolution in Onobrychis (Fabaceae)
Source: Int J Mol Sci. 2022 Sep 20;23(19):11033. doi: 10.3390/ijms231911033 (PMC9570107; doi:10.3390/ijms231911033)
Supplement: Supplementary file 1 [file ijms-23-11033-s001.zip › ijms-1927409-supplementary.pdf]

Supplementary Materials

Title: The Chromosome Number and rDNA Loci Evolution in *Onobrychis* (Fabaceae)
Authors: Gulru Yucel, Alexander Betekhtin, Evren Cabi, Metin Tuna Robert Hasterok, Bozena Kolano

The following Supplementary Materials are available for this article:

Table S1. Chromosome numbers (2*n*) of *Onobrychis* species analysed in the present study and data retrieved from literature.

Species	Chromosome number – the present study	Chromosome number - literature data
O. alba subsp. laconica	14	14 [56]
O. altissima	28	14 [37]<br>28 [6,45]
O. arenaria	28	14 [47]<br>28[47]
O. biebersteinii	28	28 [48]
O. caput galli	14	14 [43]<br>28 [7]
O. chorossanica	16	14 [10]
O. crista-galli	16	16 [6]<br>32 [7]
O. cyri	28	28 [49]
O. gaubae	16	16 [6]
O. gracilis	14	14 [8]<br>28 [56]
O. grandis	14	14 [50]
O. humilis	14	14 [51]
O. hypargyrea	14	14 [8,43]
O. iberica	16	28 [52]
O. inermis	28	28 [48]
O. kachetica	16	16 53]
O. megataphros	14	32 [9]
O. michauxii	16	14 [10]<br>16 [10]
O. persica	16	14 [45]<br>28 [6]
O. ptolemaica	14	14 [10]<br>16 [7]
O. radiata	16	14 [45]
O. sintenisii	16	14 [10]
O.stenorhiza	14	-
O. supina	14	14 [54]
O. subacaulis	32	16 [37]
O. transcaucasica	28	28 [49]
O. vaginalis	16	-
O. vassilczenkoi	16	14 [55]
O. viciifolia	28	28 [6]

Table S2. The taxonomic classification of *Onobrychi*s species.

**Sırjaev [4]**	**Grossheim [89]**	**Rechinger [90]**
**Subgenus *Onobrychis***	**Subgenus *Onobrychis***	**Subgenus *Onobrychis***
**Section *Onobrychis***	**Section *Onobrychis***	**Section *Onobrychis***
O. megataphros	*O. biebersteinii*	*O .megataphros*
O. gracilis	*O. gracilis*	*O. transcaucasica*
O. biebersteinii	*O. cyri*	*O. altissima*
O. arenaria	*O. viciifolia*	*O. persica*
O. viciaefolia	*O. transcaucasica*	
O. supina	*O. altissima*	
O. alba	*O. inermis*	
O. inermis	*O. iberica*	
O. humilis	*O. arenaria*	
O. stenorhiza		
**Section *Lophobrychis***	**Section *Lophobrychis***	**Section *Lophobrychis***
O. caput-galli	*O. caput-galli*	*O. caput-galli*
O. crista-galli		*O. crista-galli*
**Subgenus *Sisyrosema***	**Subgenus *Sisyrosema***	**Subgenus *Sisyrosema***
**Section *Hymenobrychis***	***Section Hymenobrychis***	***Section Hymenobrychis***
O. sintenisii	*O. sintenisii*	*O. sintenisii*
O. chorossanica	*O. chorossanica*	*O. chorossanica*
O. michauxii	*O. michauxii*	*O. michauxii*
O. radiata	*O. vassilczenkoi*	*O. ptolemaica*
O. ptolemaica	*O. vaginalis*	
O. hypargyrea	*O. kachetica*	
	O.radiata	
**Section *Anthyllium***	**Section *Anthyllium***	**Section *Anthyllium***
O. grandis	*O. grandis*	*O. grandis*
**Section *Heliobrychis***	**Section *Heliobrychis***	**Section *Heliobrychis***
O. subacaulis	*O. subacaulis*	*O. subacaulis*
		O. gaubae

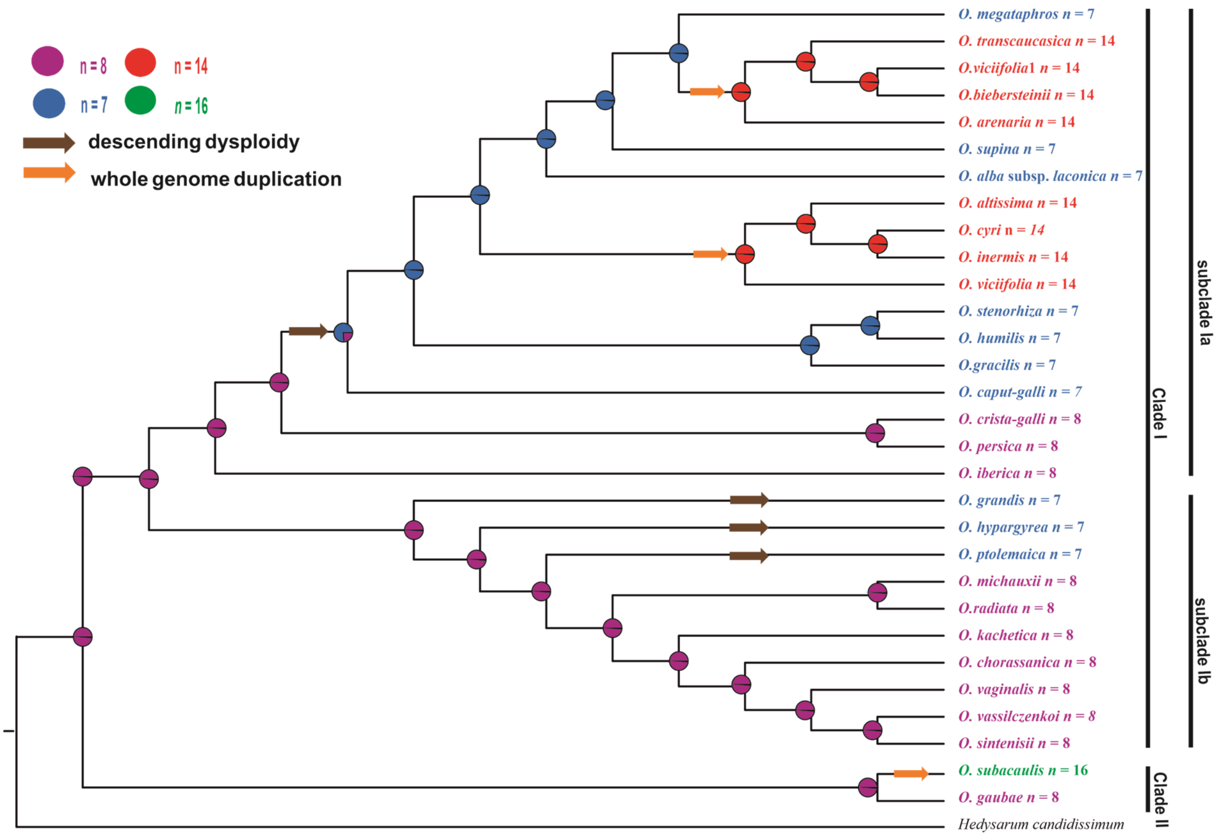

Figure S1. Ancestral state reconstruction of the haploid chromosome numbers of the analysed species of *Onobrychis*. The chromosome numbers have been mapped on the ML tree of nrITS sequences using the maximum likelihood method implemented in ChromEvol 2.0 software. The tree was rooted with *Hedysarum candidissimum*.
